# Supplementary material for: Silencing LGR6 Attenuates Stemness and Chemoresistance via Inhibiting Wnt/β-Catenin Signaling in Ovarian Cancer
Source: Mol Ther Oncolytics. 2019 Apr 19;14:94–106. doi: 10.1016/j.omto.2019.04.002 (PMC6517611; doi:10.1016/j.omto.2019.04.002)
Supplement: Document S1. Tables S1–S6 [file mmc1.pdf]

OMTO, Volume *14*

Supplemental Information

Silencing LGR6 Attenuates Stemness and Chemoresistance via Inhibiting Wnt/β-Catenin Signaling in Ovarian Cancer

Xiaohong Ruan, Aibin Liu, Meigong Zhong, Jihong Wei, Weijian Zhang, Yingrou Rong, Wanmin Liu, Mingwei Li, Xingrong Qing, Gaowen Chen, Ronggang Li, Yuehua Liao, Qiongru Liu, Xin Zhang, Dong Ren, and Yifeng Wang

Supplemental Table 1. The basic information of 40 patients with normal ovarian control for LGR6 immunohistochemical staining analysis.

		Cases (n, %)
Age	<50	11 (27.5)
	≥50	29 (72.5)
Type of diseases	Malignant lesions on the contralateral ovary	1 (2.5)
	Non-malignant lesions on the contralateral ovary	13 (32.5)
	Malignant lesions without ovary	5 (12.5)
	Non-malignant lesions without ovary	21 (52.5)

Note: (1) Malignant lesions on the contralateral ovary included endometrioid adenocarcinoma. (2) Non-malignant lesions on the contralateral ovary included simple cyst, Brenner tumor, serous cystadenoma and fibrothecoma. (3) Malignant lesions without ovary included breast cancer, cervical cancer and uterus cancer. (4) Non-malignant lesions without ovary included cervical intraepithelial neoplasia, mesosalpinx cyst, uterine fibroids, endometrial hyperplasia with atypia and prolapse of uterus.

Supplemental Table 2. The basic information of 294 patients with epithelial ovarian cancer for LGR6 immunohistochemical staining analysis.

		Cases (n)	Percentage (%)
Age	<50	128	43.5
	≥50	166	56.5
Histologic	Low grade serous adenocarcinoma	27	9.2
	High grade serous adenocarcinoma	162	55.1
	Mucinous adenocarcinoma	42	14.3
	Endometrioid adenocarcinoma	29	9.9
	Clear cell adenocarcinoma	16	5.4
	Other	18	6.1
FIGO stage	Stage I	90	30.6
	Stage II	49	16.7
	Stage III	130	44.2
	Stage IV	12	4.1
	NA	13	4.4
Progression status (in 3 y)	Positive	91	31.0
	Negative	54	18.4
	NA	149	50.7
First chemotherapy response (at 6 m)	Sensitivity	63	21.4
	Resistance	30	10.2
	NA	201	68.4

Note: (1) Other histologic types included mixed adenocarcinoma, serous-mucinous adenocarcinoma, undifferentiated carcinoma, carcinosarcoma and non-specific type adenocarcinoma. (2) NA: Not available; FIGO: The International Federation of Gynecology and Obstetrics.

Supplemental Table 3. The relationship between LGR6 IHC expression level and clinical pathological characteristics in 294 patients with epithelial ovarian cancer.

Parameters	Number of cases	LGR6 IHC expression		P values
		Low	High	
Age				
<50	128	55	63	0.705
≥50	166	86	90	
Histologic				
Type I	114	68	46	<0.001
Type II	164	62	102	
FIGO stage				
I – II	139	84	55	<0.001
III - IV	142	49	93	
Progression status (in 3 y)				
Positive	91	20	70	0.004
Negative	54	24	29	
First chemotherapy response (at 6 m)				
Sensitivity	63	39	24	<0.001
Resistance	30	2	28	

Note: (1) Histologic type I included low grade serous adenocarcinoma, mucinous adenocarcinoma, endometrioid adenocarcinoma and clear cell adenocarcinoma. Histologic type II included high grade serous adenocarcinoma and undifferentiated carcinoma. (2) IHC: Immunohistochemistry; FIGO: The International Federation of Gynecology and Obstetrics.

Supplemental Table 4. A list of primers used in the reactions for real-time PCR.

Gene name		Sequence
ABCG2	forward	5'- ATGAACACACATGTGCAACCATC -3'
	reverse	5'- CACAGAAACACAACACTTGGCTGTA -3'
CCND1	forward	5'- CGTGGCCTCTAAGATGAAGG -3'
	reverse	5'- CCACTTGAGCTTGTTCACCA -3'
CDH1	forward	5'- ATGAGTGTCCCCCGGTATCT -3'
	reverse	5'- GGCTGTGGGGTCAGTATCAG -3'
DKK3	forward	5'- GTGCTGTGCCTTCCAGAGA -3'
	reverse	5'- GCTCTAGCTCCCAGGTGATG -3'
GAPDH	forward	5'- GCACCGTCAAGGCTGAGAAC -3'
	reverse	5'- TGGTGAAGACGCCAGTGGA -3'
KLF4	forward	5'- GTTCCCATCTCAAGGCACAC -3'
	reverse	5'- ACGGTAGTGCCTGGTCAGTT -3'
LEF1	forward	5'- CCATCCCGAGAACATCAAAT -3'
	reverse	5'- CCTGGAGAAAAGTGCTCGTC -3'
LGR4	forward	5'- TGATGCAGCAAATGTCACAA -3'
	reverse	5'- GGGCTTAAAAGCACCTGTTG -3'
LGR5	forward	5'- TGAATTTGGCTTGGAACAAA -3'
	reverse	5'- AAAGACGACAGGAGGTTGGA -3'
LGR6	forward	5'- AAGGGAACCTTGCTCTCTCC -3'
	reverse	5'- CATCCCATAGGGACAGCACT -3'
MMP7	forward	5'- TGTATGGGGAACTGCTGACA -3'
	reverse	5'- ATCTCCTCCGAGACCTGTCC -3'
NANOG	forward	5'- TCCAACATCCTGAACCTCAGCTA -3'
	reverse	5'- AGTCGGGTTCACCAGGCATC -3'
OCT4	forward	5'- TGAAGCTGGAGAAGGAGAAGCTG -3'
	reverse	5'- GCAGATGGTCGTTTGGCTGA -3'
SOX2	forward	5'- GTGAGCGCCCTGCAGTACAA -3'
	reverse	5'- GCGAGTAGGACATGCTGTAGGTG -3'
TCF1	forward	5'- ACCTGTCCCAACACCTCAAC -3'
	reverse	5'- CTCATCACCTGTGGGCTCTT -3'
VEGFA	forward	5'- AGACCCTGGTGGACATCTTC -3'
	reverse	5'- ATCCGCATAATCTGCATGGT -3'

Supplemental Table 5. A list of primers used in the reactions for clone PCR.

Gene name		Sequence
shLGR6:#1	forward	5'- CCGG-GAGGGAAGTAAAGACAGTGAA-CTCGAG-TTCACTGTCTTTACTTCCCTC-TTTTTG -3'
	reverse	5'-CAAAAA-GAGGGAAGTAAAGACAGTGAA-CTCGAG-TTCACTGTCTTTACTTCCCTC-CCGG-3'
shLGR6#2	forward	5'- CCGG-GCATTCCAGTACCTGCCTAAA-CTCGAG-TTTAGGCAGGTACTGGAATGC-TTTTTG -3'
	reverse	5'- CAAAAA-GCATTCCAGTACCTGCCTAAA-CTCGAG-GCATTCCAGTACCTGCCTAAA-CCGG-3'

Supplemental Table 6. The basic information of 6 epithelial ovarian cancer patients and 4 normal ovarian control patients for LGR6 mRNA and protein expression analysis.

		Cases (n, %)	
		EOC	NC
Age	≤50	3 (50.0)	2 (50.0)
	>50	3 (50.0)	2 (50.0)
Disease	Low grade serous adenocarcinoma	1 (16.7)	0
	High grade serous adenocarcinoma	2 (33.3)	0
	Mucinous adenocarcinoma	1 (16.7)	0
	Endometrioid adenocarcinoma	1 (16.7)	0
	Clear cell adenocarcinoma	1 (16.7)	0
	Cervical adenocarcinoma	0	2 (50.0)
	Endometrial adenocarcinoma	0	1 (50.0)
	Cervical squamous carcinoma	0	1 (50.0)

Note: EOC: epithelial ovarian cancer; NC: normal control.
