# Supplementary material for: Silencing LGR6 Attenuates Stemness and Chemoresistance via Inhibiting Wnt/β-Catenin Signaling in Ovarian Cancer
Source: Mol Ther Oncolytics. 2019 Apr 19;14:94–106. doi: 10.1016/j.omto.2019.04.002 (PMC6517611; doi:10.1016/j.omto.2019.04.002)
Supplement: Document S2. Article plus Supplemental Information [file mmc2.pdf]

Molecular Therapy
Oncolytics
Original Article

Silencing LGR6 Attenuates Stemness and Chemoresistance via Inhibiting Wnt/β-Catenin Signaling in Ovarian Cancer

Xiaohong Ruan,[1,2] Aibin Liu,[3,4] Meigong Zhong,[5] Jihong Wei,[2] Weijian Zhang,[2] Yingrou Rong,[2] Wanmin Liu,[2] Mingwei Li,[2] Xingrong Qing,[2] Gaowen Chen,[1] Ronggang Li,[6] Yuehua Liao,[6] Qiongru Liu,[6] Xin Zhang,[7,8,9] Dong Ren,[7,8] and Yifeng Wang[1]

[1]Department of Obstetrics and Gynecology, Zhujiang Hospital, Southern Medical University, Guangzhou 510282, People's Republic of China; [2]Department of Gynecology, Jiangmen Central Hospital, Affiliated Jiangmen Hospital of Sun Yat-sen University, Jiangmen 529030, People's Republic of China; [3]Department of Geriatrics, Xiangya Hospital, Central South University, Changsha 410008, People's Republic of China; [4]National Clinical Research Center for Geriatric Disorders, Xiangya Hospital, Central South University, Changsha 410008, People's Republic of China; [5]Department of Pharmacy, Jiangmen Maternity and Child Health Care Hospital, Jiangmen 529030, China; [6]Department of Pathology, Jiangmen Central Hospital, Affiliated Jiangmen Hospital of Sun Yat-sen University, Jiangmen 529030, People's Republic of China; [7]Clinical Experimental Center, Jiangmen Key Laboratory of Clinical Biobanks and Translational Research, Jiangmen Central Hospital, Affiliated Jiangmen Hospital of Sun Yat-sen University, Jiangmen 529030, China; [8]Dongguan Key Laboratory of Medical Bioactive Molecular Developmental and Translational Research, Guangdong Provincial Key Laboratory of Medical Molecular Diagnostics, Guangdong Medical University, Dongguan 523808, China; [9]Collaborative Innovation Center for Antitumor Active Substance Research and Development, Guangdong Medical University, Zhanjiang, Guangdong 524023, China

Leucine-rich-repeat-containing G protein-coupled receptors (LGRs) have been widely found to be implicated with development and progression in multiple cancer types. However, the clinical significance and biological functions of LGR6 in ovarian cancer remains unclear. In this study, LGR6 expression was mainly examined by immunohistochemistry. Functional assays *in vitro* and animal experiments *in vivo* were carried out to explore the effect of LGR6 on cancer stem cell (CSC) characteristics and chemotherapeutic responses in ovarian cancer cells. Luciferase assays and GSEA were used to discern the underlying mechanisms contributing to the roles of LGR6 in ovarian cancer. Here, we reported that LGR6 was upregulated in ovarian cancer, which positively correlated with poor chemotherapeutic response and progression survival in ovarian cancer patients. Loss-of-function assays showed that downregulating LGR6 abrogated the CSC-like phenotype and chemoresistance *in vitro*. More importantly, silencing LGR6 improved the chemoresistance of ovarian cancer cells to cisplatin *in vivo*. Mechanistic investigation further revealed that silencing LGR6 inhibited stemness and chemoresistance by repressing Wnt/β-catenin signaling. Collectively, our results uncover a novel mechanism contributing to LGR6-induced chemotherapeutic resistance in ovarian cancer, providing the evidence for LGR6 as a potential therapeutic target in ovarian cancer.

INTRODUCTION

Ovarian cancer is one of the most common gynecological malignancies, as well as one of the leading causes responsible for the cancer-related deaths in females.[1] Despite substantial improvements in treatment of ovarian cancer in the past several decades, the prognosis of ovarian cancer patients is still dismal, which is largely attributed to chemotherapeutic resistance after a long period of treatment. Cancer stem cells (CSCs) that are a minority population of cells with the abilities of unrestrained proliferation and self-renewal have been identified to contribute to the failure of chemotherapy in ovarian cancer patients.[2,3] Several lines of evidence have reported that CSCs are crucial mediators in the induction and maintenance of chemotherapeutic resistance in several human cancers,[4,5] including ovarian cancer.[6,7] Thus, identification of the underlying mechanisms that induce and maintain CSC properties will heighten the efficacy of chemotherapeutics and improve prognosis in ovarian cancer patients.

Wnt/β-catenin signaling pathways are a group of signal transduction pathways consisting of several ligands and receptor proteins that passed transduction signal from outside the cell to the inside. The ligands binding to receptors trigger signaling pathways that play a crucial role in embryonic development and tissue regeneration.[8,9] The constitutive activation of Wnt/β-catenin signaling has been extensively identified to be implicated in multifaceted aspects of cancers, including cancer dormancy, metastasis, and progression.[10,11] Furthermore, mounting studies have reported that sustained activation of canonical Wnt/β-catenin signaling is pivotal in inducing and maintaining CSC features in various types

Received 13 January 2019; accepted 10 April 2019;
https://doi.org/10.1016/j.omto.2019.04.002.

Correspondence: Yifeng Wang, Department of Obstetrics and Gynecology, Zhujiang Hospital, Southern Medical University, Guangzhou 510282, People's Republic of China.
E-mail: wyf2015@163.com

This is an open access article under the CC BY license (http://creativecommons.org/licenses/by/4.0/).

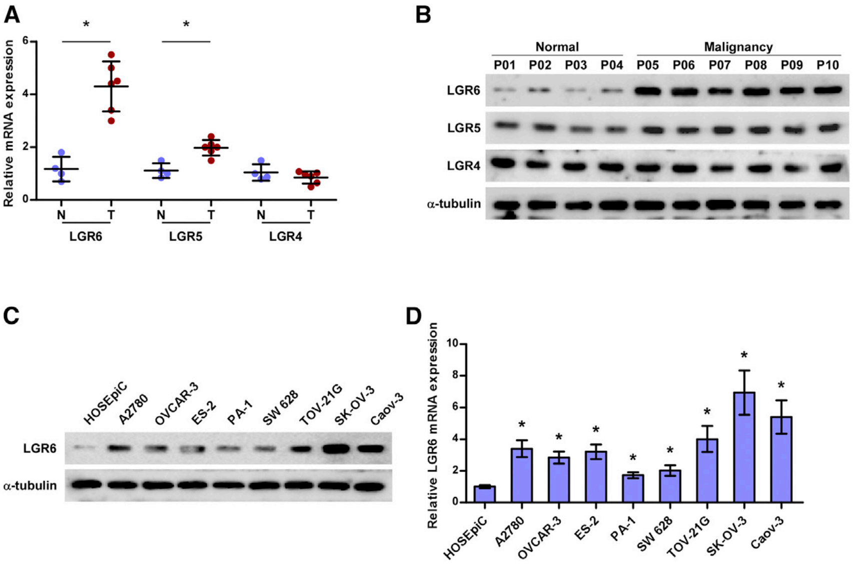

Figure 1. LGR6 Is Upregulated in Ovarian Cancer

(A) LGR4–6 expression in six ovarian cancer tissues and four normal ovarian epithelial tissues by real-time PCR. Each bar represents the median values ± quartile values. *$p < 0.05$. (B) LGR4–6 expression in six ovarian cancer tissues and four normal ovarian epithelial tissues by western blotting. (C and D) Western blotting (C) and real-time PCR (D) of LGR6 expression in one normal ovarian epithelial cell HOSEpiC and eight ovarian cancer cell lines. GAPDH was used as an endogenous control in RT-PCR, and α-tubulin was detected as a loading control in the western blot. Error bars represent the mean ± SD of three independent experiments. *$p < 0.05$.

of cancer.[12–14] Likewise, Wnt/β-catenin signaling has been reported to be required for CSCs in ovarian cancer. Chen et al.[15] has found that STAT3 was found to be hyperactivated in ovarian cancer spheroids, where activity of Wnt/β-catenin was indispensable for STAT3-induced or maintained stemness in ovarian cancer cells; moreover, Mariya and colleagues[16] have reported that MMP10 promoted stemness and chemotherapeutic resistance of ovarian cancer stem-like cells by activating Wnt/β-catenin signaling. Therefore, further elucidating the mechanisms responsible for constitutive activation of Wnt signaling in ovarian cancer is of paramount importance.

Leucine-rich-repeat-containing G protein-coupled receptor (LGR) is a subgroup of the seven-transmembrane G protein-coupled superfamily and is well-known for the member proteins LGR4–6.[17] Much research efforts have been recently made to explore the biological functions of LGR4–6 in multiple human cancer types.[18–21] Interestingly, numerous studies have reported that LGR4–6 play crucial roles in activation of Wnt/β-catenin signaling via binding to R-spondins (Rspo1–4).[22–24] However, the clinical significances and biological roles of LGR4–6 in ovarian cancers, as well as the regulatory functions of LGR4–6 on Wnt/β-catenin signaling in the context of ovarian cancers, have yet not to be elucidated. Here, our results reported that LGR6, not LGR4 or LGR5, was dramatically upregulated in ovarian cancer tissues, and overexpression of LGR6 significantly correlated with poor clinicopathological characteristics, as well as predicted poor overall and progression-free survival in ovarian cancer patients. Loss-of-function assays showed that silencing LGR6 repressed the stemness and improved chemoresistance of ovarian cancer cells. Mechanistic investigation further revealed that LGR6 promoted stemness and chemoresistance via Wnt/β-catenin signaling in ovarian cancer cells. Thus, our results indicate that LGR6 might be used as a potential therapeutic target in ovarian cancer.

RESULTS

LGR6 Is Upregulated in Ovarian Cancer

To determine the expression levels of different members of the LGR family in ovarian cancer, six ovarian cancer tissues and four normal ovarian tissues were collected, and LGR4, LGR5, and LGR6 were further examined in these tissues by real-time PCR and western blot. As shown in Figures 1A and 1B, mRNA and protein levels of LGR6 were dramatically upregulated, and LGR5 was slightly increased in ovarian cancer tissues compared with those in normal ovarian tissues, but there was no significant difference of LGR4 expression between in ovarian cancer tissues and normal ovarian tissues. We further examined the expression levels of LGR6 in one ovarian epithelial cell line, HOSEpiC, and eight ovarian cancer cells, respectively, and found that LGR6 expression was differentially enhanced in ovarian cancer cells compared with that in HOSEpiC (Figures 1C and 1D). These results demonstrated that high levels of LGR6 may be implicated in the pathogenesis of ovarian cancer.

Overexpression of LGR6 Predicts Poor Prognosis and Progression

We further determined the clinical significance of LGR6 in different histologic types of ovarian cancer by immunohistochemistry (IHC) (Tables S1 and S2). As shown in Figures 2A–2C, LGR6 expression levels were robustly and significantly upregulated in different histologic types of ovarian cancer, including serous adenocarcinoma, mucinous adenocarcinoma, endometrioid adenocarcinoma, and clear-cell adenocarcinoma, compared with those in normal ovarian epithelial tissues, particularly in high-grade serous adenocarcinoma, and high expression of LGR6 was detected in 153/294 ovarian cancer tissues (52.0%). The correlation of LGR6 with clinicopathological features in ovarian cancer patients were further investigated, and the results showed that high expression of LGR6 positively correlated with histologic types, International Federation of Gynecology and Obstetrics (FIGO) stages, poor chemotherapeutic response, and poor progression in ovarian cancer patients (Figures 2D and 2E; Table S3). Kaplan-Meier survival analysis indicated that overexpression of LGR6 was significantly associated with poor progression-free survivals in ovarian cancer patients (Figure 2F). The analysis result of

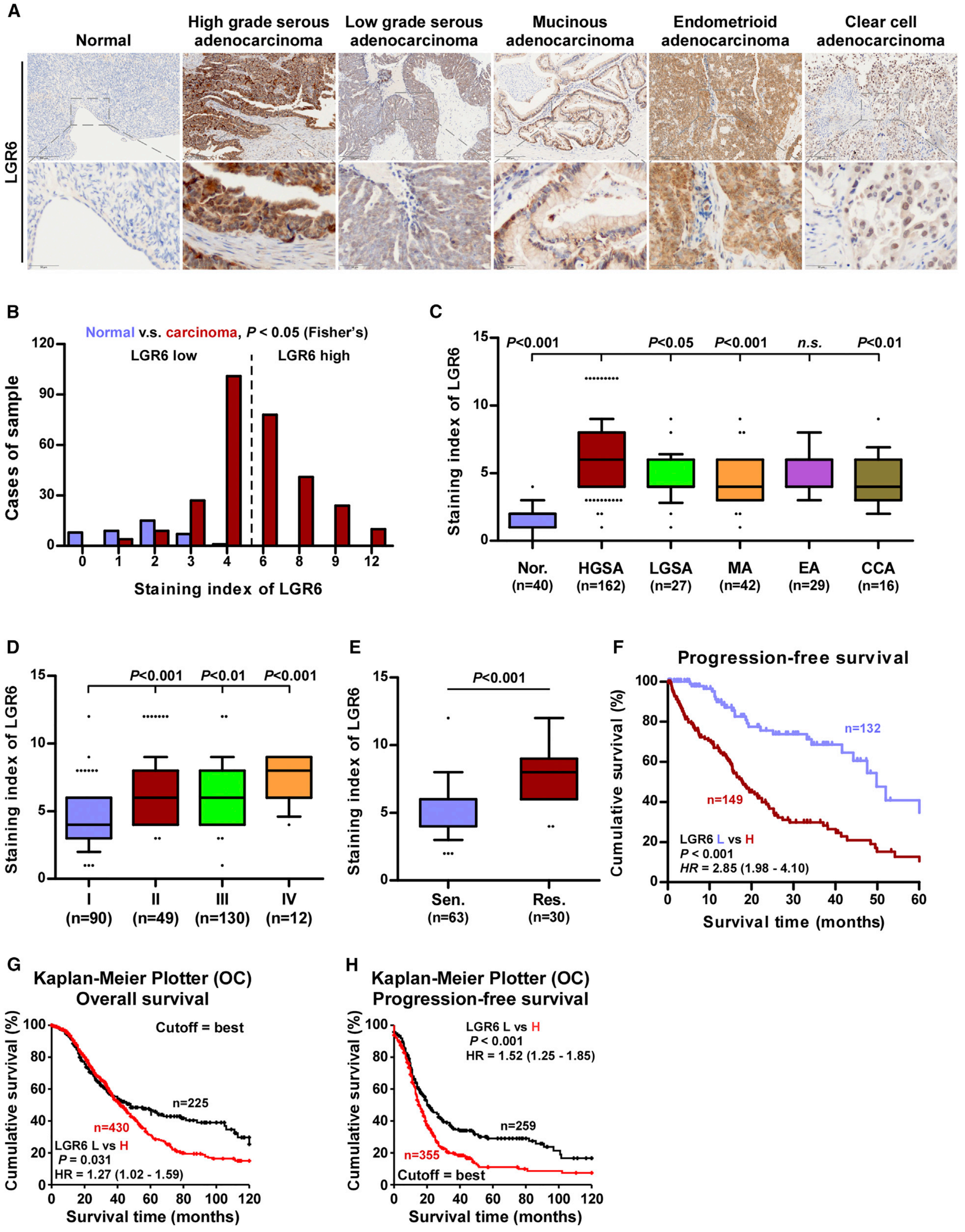

(legend on next page)

the ovarian cancer dataset from Kaplan-Meier plotter further revealed that high expression of LGR6 predicted poorer overall and progression-free survival in ovarian cancer patients (Figures 2G and 2H). Therefore, these results indicated that the high expression of LGR6 is closely associated with poor prognosis and disease progression in ovarian cancer patients.

Silencing LGR6 Represses Stemness in Ovarian Cancer

The above-mentioned findings indicated that high levels of LGR6 significantly contributed to poor progression in ovarian cancer patients. Accumulating evidence has shown that existence of CSCs promote the early progression and recurrence in a variety of cancers.[25,26] Therefore, we further investigated the effects of LGR6 on the CSC phenotypes of ovarian cancer cells. We first constructed stable LGR6-downexpressing cell lines by endogenously knocking down LGR6 via retrovirus infection in SK-OV-3 and Caov-3 cells that expressed the highest levels of LGR6 in all ovarian cancer cells (Figures 3A and 3B). Cell counting kit-8 (CCK-8) assay showed that silencing LGR6 had no significant effect on the proliferation rate of SK-OV-3 and Caov-3 cells (Figure 3C). Spheroid formation assay was performed first, and the results showed that silencing LGR6 suppressed spheroid formation ability in ovarian cancer cells (Figure 3D). Side population (SP) analysis showed that downregulating LGR6 reduced the fraction of SP cells (Figure 3E). The $CD133^+$ population of ovarian cancer cells was repressed by silencing LGR6 via flow cytometry (Figure 3F). The effects of LGR6 on expression levels of stem-cell factors, including ABCG2, OCT4, SOX2, NANOG, and KLF4, were further examined via RT-PCR, and the results showed that silencing LGR6 reduced the expression of these factors (Figure 3G). These results demonstrated that silencing LGR6 repressed CSC characteristics in ovarian cancer cells.

Silencing LGR6 Attenuates Chemoresistance of Ovarian Cancer Cells

The previous results indicated that overexpression of LGR6 was positively correlated with poor chemotherapeutic response in ovarian cancer patients, to which CSCs have been reported to contribute.[6,7] Therefore, we further examined the effects of LGR6 on therapeutic response of ovarian cancer cells to different chemotherapies commonly used in ovarian cancer patients. Cell-viability assays showed that silencing LGR6 decreased the viability of ovarian cancer cells under treatment of cisplatin or paclitaxel (Figure 4A). Similarly, silencing LGR6 dramatically enhanced the apoptosis rate of ovarian cancer cells treated with cisplatin or paclitaxel (Figure 4B). Mitochondrial membrane potential assay revealed that silencing LGR6 inhibited the mitochondrial potential of ovarian cancer cells after treatment of cisplatin or paclitaxel (Figure 4C). The effect of silencing LGR6 on the expression of anti-apoptotic proteins Bcl-2 and Bcl-xL and caspase-3 and -9 activity was further examined. As shown in Figures 4D–4F, silencing LGR6 elevated the activity of caspase-3 and -9 but reduced the expression of Bcl-2 and Bcl-xL in ovarian cancer cells. Collectively, these results indicate that silencing LGR6 abrogates chemoresistance in ovarian cancer cells.

Silencing LGR6 Improves Chemoresistance *In Vivo*

We further investigated the effects of LGR6 on the chemoresistance of ovarian cancer cells *in vivo*. Mice were randomly divided into two groups (n = 5/group). The SK-OV-3 vector or LGR6 sh#1 cells were inoculated subcutaneously into two groups in the left dorsal flank, respectively. Then, both groups of mice were intraperitoneally injected with cisplatin (2 mg/kg.day) every 5 days after 12 days of cells inoculation (Figure 5A). As shown in Figures 5A–5C, the tumor volumes and weight in the mice injected with the LGR6 sh#1 cells were dramatically reduced compared to those in the vector group. Furthermore, LGF6 expression levels were downregulated in the tumor tissues of the mice injected with the LGR6 sh#1 cells at the end of the experiment (Figure 5D); conversely, caspase-3 and -9 activity were remarkably elevated (Figures 5E and 5F). Thus, these findings indicated that silencing LGR6 re-sensitizes ovarian cancer cells to cisplatin *in vivo*.

Silencing LGR6 Inhibits Canonical Wnt Signaling

To uncover the mechanism underlying the effect of LGR6 on chemoresistance in ovarian cancer, gene set enrichment analysis (GSEA: http://software.broadinstitute.org/gsea/index.jsp) of LGR6 expression against the oncogenic signatures collection of the MSigDB (http://software.broadinstitute.org/gsea/msigdb/index.jsp) was performed. As shown in Figure 6A, LGR6 expression level was significant and positively correlated with the activity of canonical Wnt signaling. Western blot analysis revealed that silencing LGR6 reduced nuclear expression of β-catenin in ovarian cancer cells (Figure 6B). TOP/FLASH activity was downregulated in LGR6-silenced ovarian cancer cells compared with that in the vector cells (Figure 6C). The effect of LGR6 downregulation on downstream target genes of Wnt/β-catenin signaling was further investigated, and the results indicated that silencing LGR6 significantly reduced the expression of multiple downstream target genes of Wnt/β-catenin signaling (Figure 6D). These results indicated that inhibition of LGR6 inhibits activity of canonical Wnt/β-catenin signaling in ovarian cancer cells.

Figure 2. High Levels of LGR6 Predicts Poor Prognosis in Ovarian Cancer Patients

(A) Representative images of LGR6 expression in normal ovarian epithelial tissues and ovarian cancer tissues with different histologic types. (B) The number of ovarian cancer tissues stratified by staining index of IHC. (C) Staining index of LGR6 in different histologic types of ovarian cancer. Error bar represents the 10th–90th percentile. (D) Staining index of LGR6 in different FIGO stages of ovarian cancer. Error bar represents the 10th–90th percentile. (E) Staining index of LGR6 in chemosensitive and chemoresistant ovarian cancer. Error bar represents the 10th–90th percentile. (F) Kaplan-Meier analysis of progression-free survival curves for ovarian cancer patients stratified by high and low expression of LGR6. (G and H) Kaplan-Meier overall survival (G) and progression-free survival (H) curves for ovarian cancer patients stratified by high and low expression of LGR6 in ovarian cancer dataset from Kaplan-Meier plotter.

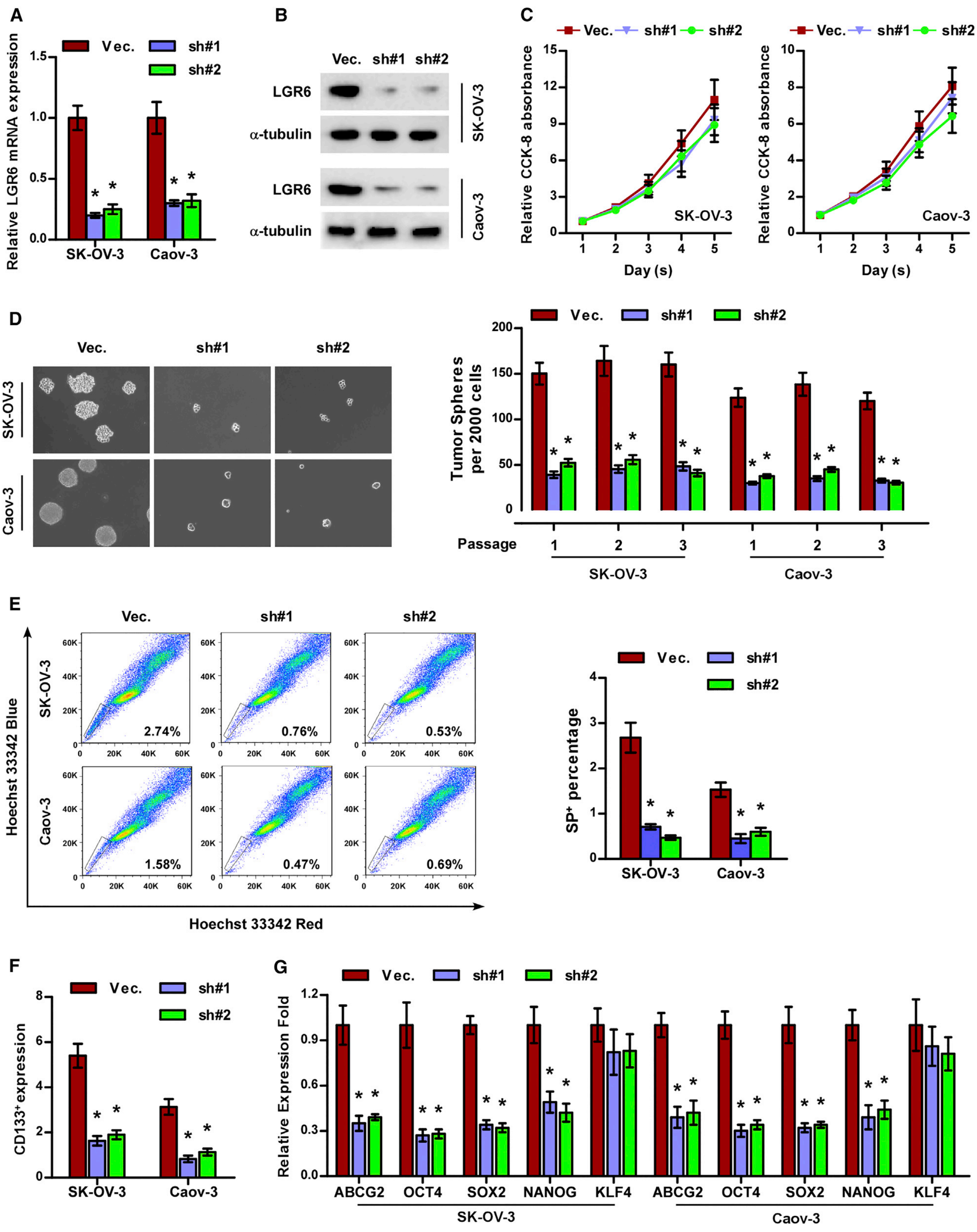

(legend on next page)

Activity of Wnt/β-Catenin Signaling Is Required for LGR6-Induced Stemness and Chemoresistance

In LGR6-silenced ovarian cancer cells, S33Y, which was used to constitutively activate Wnt/β-catenin signaling as a mutant-β-catenin (serine 33 to tyrosine),[27] was further transfected to investigate the functional role of Wnt/β-catenin signaling in the regulatory functions of LGR6 in stemness and chemoresistance in ovarian cancer cells. First, S33Y significantly reversed activity of Wnt/β-catenin signaling repressed by LGR6 downregulation in ovarian cancer cells (Figure 7A). Spheroid formation assay and flow cytometry showed that S33Y significantly enhanced the stemness in LGR6-silenced cells (Figures 7B–7D). Consistently, the chemoresistant ability of the LGR6-silenced ovarian cancer cells to cisplatin or paclitaxel were dramatically elevated by S33Y (Figure 7E). Therefore, these results indicated that silencing LGR6 inhibits stemness and chemoresistance of ovarian cancer cells via repressing canonical Wnt/β-catenin signaling.

DISCUSSION

The pivotal findings of this study provide insights into the functional role of LGR6 in ovarian cancer. In the present manuscript, our results demonstrated that LGR6 was upregulated in ovarian cancer tissues, which positively correlated with shorter overall and progression-free survival in ovarian cancer patients. In addition, silencing LGR6 not only inhibited CSC-like phenotypes, but also attenuated the chemoresistance of ovarian cancer cells *in vitro* and *in vivo*. Our results further revealed that silencing LGR6-induced suppression of stemness and chemoresistance in ovarian cancer tissues was dependent on Wnt/β-catenin signaling. Therefore, our findings unravel a novel mechanism by which LGR6 promotes the CSC characteristics and chemoresistance in ovarian cancer cells.

LGR6 has been widely reported to serve as an important stem cell marker in multiple cancer types, which significantly contributed to carcinogenesis and progression of cancer.[28,29] Paradoxically, two literatures have reported that LGR6 potentially functioned as a tumor suppressor in colon cancer and breast cancer.[24,30] These findings suggest that the pro- and anti-tumor roles of LGR6 are tumor type dependent. In ovarian cancer, LGR6 has been found to be associated with the development and progression of high-grade serous ovarian carcinoma.[31] However, the clinical significance and functional role of LGR6 in ovarian cancer remains not reported yet. In this study, our results reported that LGR6 was differentially upregulated in different histologic types of ovarian cancer, particularly in high-grade serous adenocarcinoma, and high expression of LGR6 positively correlated with histologic types, FIGO stages, poor chemotherapeutic response, and poor progression in ovarian cancer patients. Furthermore, functional experiments showed that silencing LGR6 inhibited CSC properties and attenuated chemoresistance in ovarian cancer cells via inactivating Wnt/β-catenin signaling. Collectively, our results determine the oncogenic role of LGR6 in ovarian cancer.

Similar to the controversial roles of LGR6 in different types of cancer, LGR proteins, including LGR4–6, have been demonstrated to play an opposite, even paradoxical, role in regulating Wnt/β-catenin signaling. Several lines of evidence have reported that LGR4–6 enhances activity of Wnt/β-catenin signaling via binding to R-spondins.[22–24] However, LGR5 was found to play a negative role in Wnt signaling in colorectal cancer,[32,33] suggesting that the exact roles of LGR4–6 in Wnt signaling and tumorigenesis appear to vary depending on the given cellular context. Notably, Wnt signaling augmented by LGR6 has been reported to support the development and progression of high-grade serous ovarian carcinoma.[31] Consistently, our findings found that silencing LGR6 robustly inhibited Wnt/β-catenin signaling in ovarian cancer cells. More importantly, repression of Wnt/β-catenin signaling by LGR6 downregulation inhibited CSC characteristics as well as enhanced the sensitivity of ovarian cancer cells to chemotherapeutics. Thus, our results provide a novel finding that LGR6 promotes stemness and chemoresistance via activating Wnt/β-catenin signaling in ovarian cancer. However, the specific mechanism underlying LGR6-induced activation of Wnt/β-catenin signaling in ovarian cancer remains unclear, which requires further investigation in the following work.

The presence of CSCs has been extensively reported to be a major contributor for the chemotherapeutic resistance in ovarian cancer. A study from Janzen et al.[6] has reported that an apoptosis-enhancing drug, birinapant, aimed at eliminating the CSC subpopulation in ovarian cancer, re-sensitized ovarian cancer cells to carboplatin via cleavage of caspase 8 and restoration of apoptosis caused by degradation of baculoviral IAP repeat containing 3 (cIAP). Furthermore, the dual prostaglandin-endoperoxide synthase/lipoxygenase (COX/LOX) inhibitor licofelone improved the efficacy of paclitaxel in ovarian cancer by suppressing tumor stem-like properties.[7] These studies have indicated that therapeutic strategy targeting CSCs is an effective avenue in improving chemoresistance in ovarian cancer. In this study, our results showed that silencing LGR6 repressed stemness in ovarian cancer cells. Importantly, inhibition of CSC-like phenotypes by LGR6 downregulation dramatically improved the chemoresistance of ovarian cancer cells to cisplatin and paclitaxel. In fact, several studies have demonstrated that LGR6 marks stem cells in

Figure 3. Silencing LGR6 Represses CSC Characteristics in Ovarian Cancer Cells *In Vitro*

(A and B) Real-time PCR (A) and western blotting (B) analysis of LGR6 expression in the indicated ovarian cancer cells. GAPDH was used as an endogenous control in RT-PCR, and α-tubulin was detected as a loading control in the western blot. Error bars represent the mean ± SD of three independent experiments. *$p < 0.05$. (C) The effect of LGR6 silencing on proliferation of ovarian cancer cells via CCK-8 assay. Error bars represent the mean ± SD of three independent experiments. (D) The effect of LGR6 silencing on spheroid formation ability of ovarian cancer cells. Error bars represent the mean ± SD of three independent experiments. *$p < 0.05$. (E and F) The effect of LGR6 silencing on SP^+ (E) and $CD133^+$ (F) population of ovarian cancer cells via flow cytometry. Error bars represent the mean ± SD of three independent experiments. *$p < 0.05$. (G) Real-time PCR of ABCG2, OCT4, SOX2, NANOG, and KLF4 expression in the indicated ovarian cancer cells. GAPDH was used as endogenous control. Error bars represent the mean ± SD of three independent experiments. *$p < 0.05$.

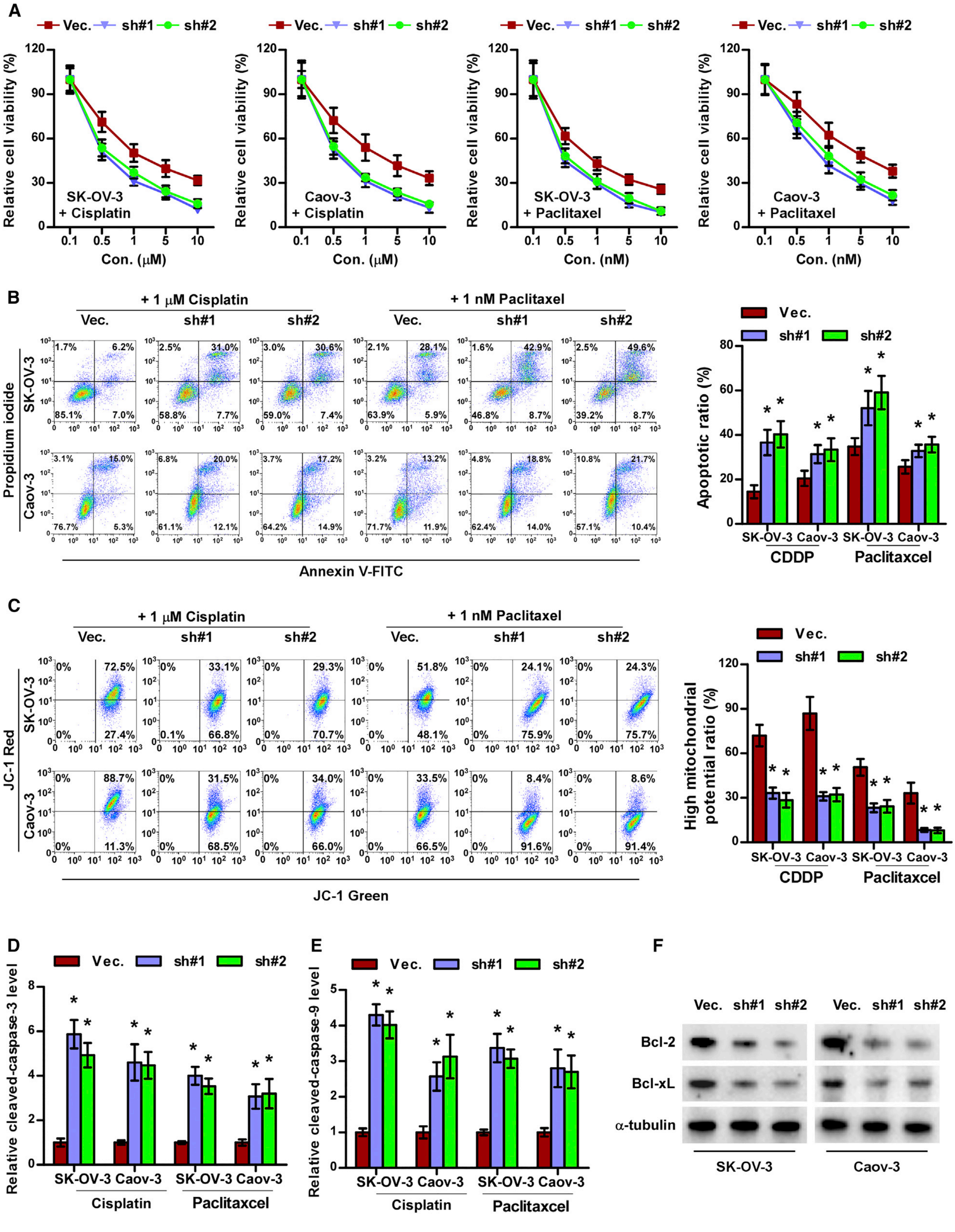

(legend on next page)

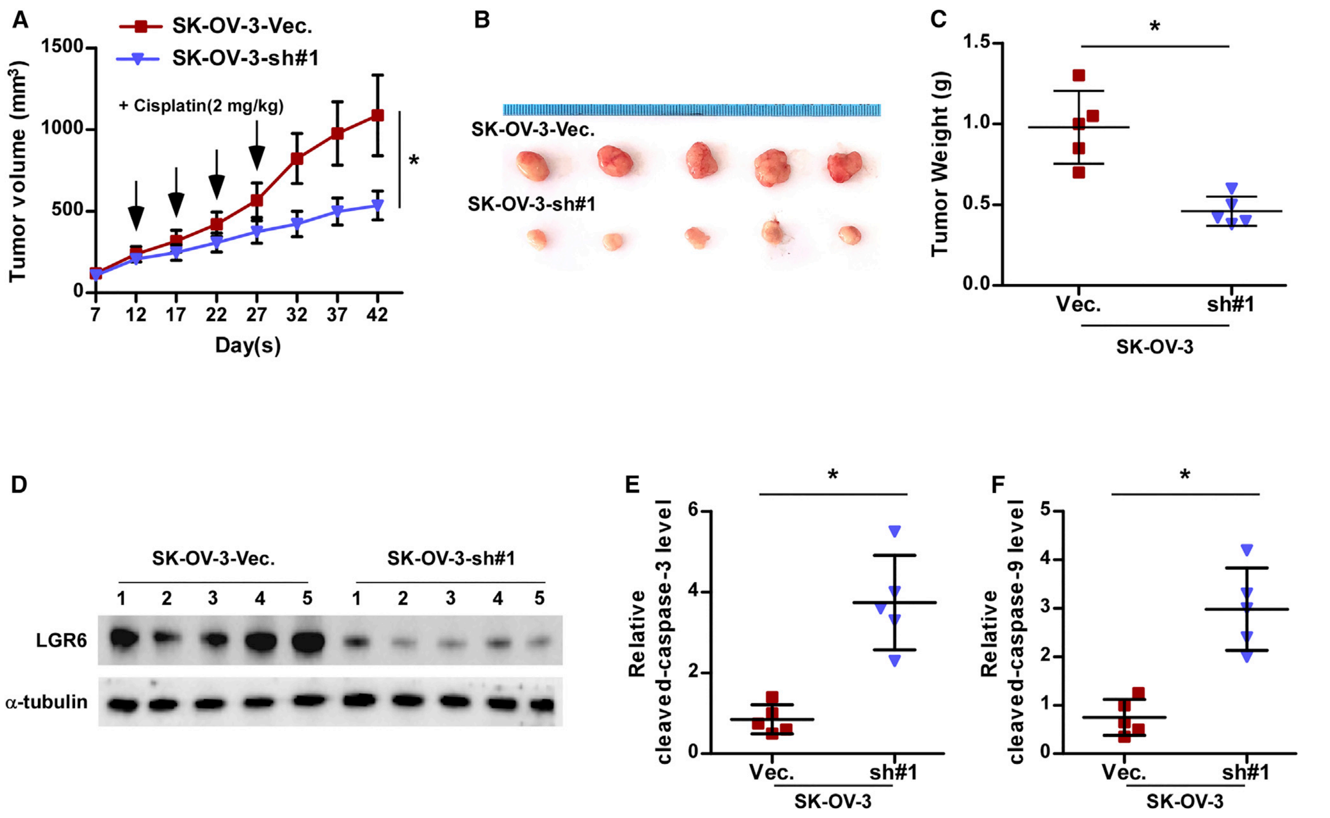

Figure 5. Silencing LGR6 Improves Chemoresistance of Ovarian Cancer Cells to Cisplatin *In Vivo*

(A) Tumor volumes were measured every 5 days in the indicated mice groups. Each bar represents the median values ± quartile values. (B) Images of excised tumors from the BALB/c mice on day 42 after injection with the indicated cells. (C) Average weight of excised tumors from the indicated mice. Each bar represents the median values ± quartile values. *$p < 0.05$. (D) Western blotting analysis of LGR6 in the indicated tumor tissues. α-tubulin served as the loading control. (E and F) Analysis of the activities of caspase-3 (E) and caspase-9 (F) in the indicated tumor tissues. Each bar represents the median values ± quartile values. *$p < 0.05$.

normal human tissues, including mammary gland,[34] skin,[35] lung,[36] and taste buds,[37] as well as in cancerous tissues,[38] providing the evidence that LGR6 serves as an important marker in maintaining stem cell properties. Therefore, our results in combination with other studies suggest that LGR6 may serve as a novel therapeutic target in the treatment of chemoresistant ovarian cancer.

In summary, our findings demonstrate that LGR6 promotes the chemoresistance of ovarian cancer cells to cisplatin via activating Wnt/β-catenin signaling pathway. Thus, better identification of the underlying mechanism responsible for the pro-chemoresistance role of LGR6 in ovarian cancer will enhance our understanding for the chemoresistance in ovarian cancer, which will facilitate the development of novel therapeutic target against ovarian cancer.

MATERIALS AND METHODS

Cell Lines and Cell Culture

The ovarian epithelial cell line HOSEpiC was purchased from ProCells, and the ovarian cancer cell lines A2780, OVCAR-3, ES-2, PA-1, SW628, TOV-21G, SK-OV-3, and Caov-3 were obtained from the Shanghai Chinese Academy of Sciences cell bank (China), and all human ovarian cancer cell lines were maintained in RPMI 1640 (Invitrogen, USA) supplemented with 10% fetal bovine serum (FBS) (HyClone, USA). All ovarian cancer cells were cultured at 37°C in a humidified atmosphere with 5% CO_2.

RNA Extraction, Reverse Transcription, and Real-Time PCR

The RNA from tissues or cells was extracted using TRIzol (Life Technologies) according to the manufacturer's instructions.

Figure 4. Silencing LGR6 Attenuates Chemoresistance in Ovarian Cancer Cells

(A) The effect of LGR6 silencing on cell viability of ovarian cancer cells. Error bars represent the mean ± SD of three independent experiments. *$p < 0.05$. (B and C) The effect of LGR6 silencing on apoptotic ratio (B) and mitochondrial potential (C) of ovarian cancer cells via flow cytometry. Error bars represent the mean ± SD of three independent experiments. *$p < 0.05$. (D and E) Analysis of the activities of caspase-3 (D) and caspase-9 (E) were detected by the cleaved forms of these two proteins. Error bars represent the mean ± SD of three independent experiments. *$p < 0.05$. (F) Western blotting analysis of Bcl-2 and Bcl-xL in the indicated cells. α-tubulin served as the loading control.

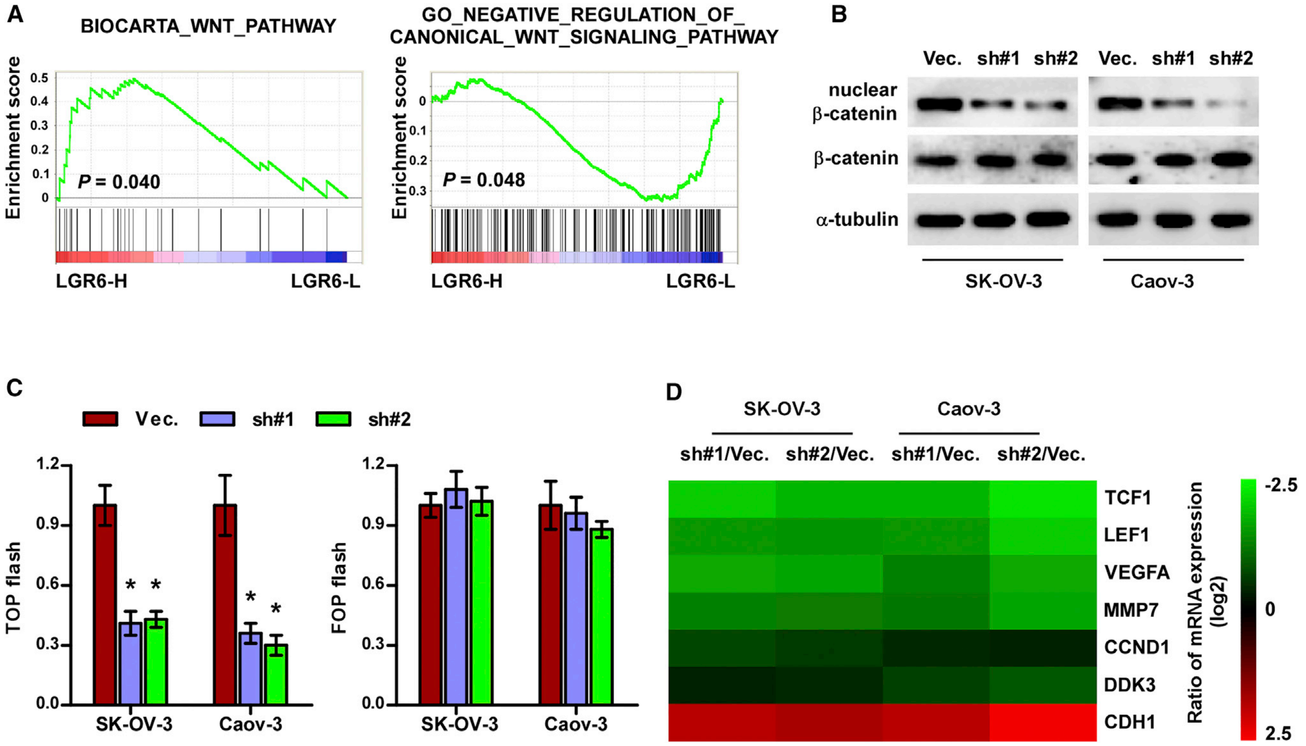

Figure 6. Silencing LGR6 Inhibits Wnt/β-Catenin Signaling

(A) GSEA analysis showed that LGR6 expression level was positively correlated with Wnt/β-catenin signaling. (B) Western blot analysis of total and nuclear expression of β-catenin in the indicated cells. p84 was used as loading control. (C) The TOP/FLASH reporter activity in the indicated ovarian cancer cells. Error bars represent the mean ± SD of three independent experiments. $^*p < 0.05$. (D) Real-time PCR analysis of TCF1, LEF1, VEGFA, MMP7, CCND1, DDK3, and CDH1 in the indicated cells.

mRNAs were polyadenylated using a poly(A) polymerase-based first-strand synthesis kit (TaKaRa, DaLian, China), and reverse transcription (RT) of total mRNA was performed using a PrimeScript RT reagent kit (TaKaRa) according to the manufacturer's protocol. cDNA was amplified and quantified on ABI 7500HT system (Applied Biosystems, Foster City, CA, USA) using SYBR green I (Applied Biosystems). The primers used in the reactions were listed in Table S4. Real-time PCR was performed as described previously.[39] Glyceraldehyde-3-phosphate dehydrogenase (GAPDH) was used as endogenous controls. Relative fold expressions were calculated with the comparative threshold cycle ($2^{-\Delta\Delta Ct}$) method as previously described.[40]

Western Blotting Analysis

Western blot was performed as described previously.[41] A cell fractionation kit (Cell Signaling Technology, USA) was used for nuclear fractionation. Antibodies against LGR6 and LGR4 were purchased from Proteintech, LGR5 and β-catenin from Invitrogen, and Bcl-2 and Bcl-xL from Cell Signaling Technology. α-tubulin antibody (Cell Signaling Technology) served as the loading control.

Immunohistochemistry

The immunohistochemistry was carried out as previously described.[42] Scores given by two independent investigators were averaged for further comparative evaluation of LGR6 expression. The proportion of tumor cells was scored as follows: 0 (no positive tumor cells); 1 (<10% positive tumor cells); 2 (10%–35% positive tumor cells); 3 (35%–70% positive tumor cells) and 4 (>70% positive tumor cells). The staining intensity score was graded according to the following criteria: 0 (no staining); 1 (weak staining, light yellow); 2 (moderate staining, yellow brown), and 3 (strong staining, brown). The staining index (SI) was calculated as the product of the staining intensity score and the proportion of positive tumor cells. Using this method of assessment, we evaluated LGR6 expression in ovarian cancer tissues by determining SI, with scores of 0, 1, 2, 3, 4, 6, 8, 9, or 12.

CCK-8 Analysis

A total of 2×10^3 cells were seeded into each well of 96 plates. The specific staining process and methods were performed as previously described.[43]

Side-Population Analysis

The cell suspensions were labeled with Hoechst 33342 (Molecular Probes, #H-3570) dye for side-population analysis as per standard protocol.[44] In brief, cells were resuspended at 1× pre-warmed OptiMEM (Gibco, USA) containing 2% FBS (Gibco, USA) at a density of 10^6/mL. Hoechst 33342 dye was added at a final concentration of 5 μg/mL in the presence or absence of verapamil (50 μmol/L; Sigma)

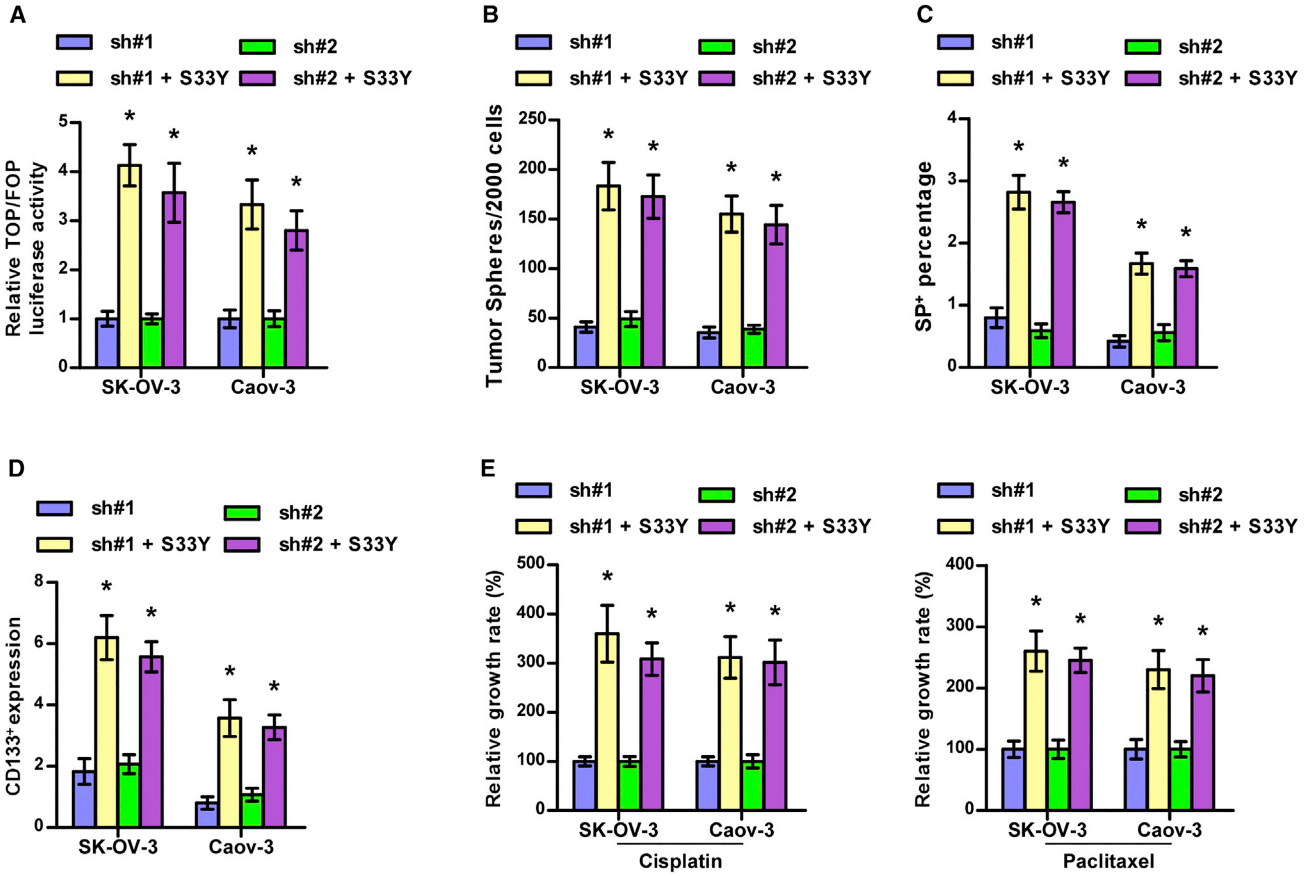

Figure 7. Silencing LGR6 Represses Stemness and Chemoresistance via Inhibiting Wnt/β-Catenin Signaling

(A) S33Y reversed the inhibitory effects of LGR6 silencing on activity of Wnt/β-catenin signaling. Error bars represent the mean ± SD of three independent experiments. $^{*}p < 0.05$. (B–D) S33Y reversed the inhibitory effects of LGR6 silencing on spheroid formation ability (B) and SP^+ (C) and $CD133^+$ (D) populations of ovarian cancer cells. Error bars represent the mean ± SD of three independent experiments. $^{*}p < 0.05$. (E) S33Y reversed the inhibitory effects of LGR6 silencing on chemoresistance of ovarian cancer cells to cisplatin and paclitaxel. Error bars represent the mean ± SD of three independent experiments. $^{*}p < 0.05$.

and the cells were incubated at 37°C for 90 min with intermittent shaking. At the end of the incubation, the cells were washed with OptiMem containing 2% FBS, centrifuged down at 4°C, and resuspended in ice-cold OptiMem containing 2% FBS and 10 mmol/L HEPES. Propidium iodide (PI, Sigma, USA) at a final concentration of 2 lg/mL was added to the cells to gate viable cells. The cells were filtered through a 40 μm cell strainer to obtain single-cell suspension before sorting. Analysis and sorting was done on a FACS AriaI (Becton Dickinson). The Hoechst 33342 dye was excited at 355 nm and its dual-wavelength emission at blue and red regions was plotted to get the SP scatter.

Spheroid Formation Assay

Cells (500 cells/well) were seeded into 6-well ultra low cluster plates (Corning) and cultured as previously described.[45] After 10–12 days, the number of cell spheroids (tight, spherical, non-adherent masses >50 μm in diameter) were counted, and images of the spheroids were scored under an inverse microscope (spheroid formation efficiency = colonies/input cells × 100%).

Flow Cytometric Analysis

Flow cytometric analysis of apoptosis used the fluorescein isothiocyanate (FITC) Annexin V apoptosis detection kit I (BD Biosciences, USA) and was presented as protocol described. In brief, cells were dissociated with trypsin and resuspended at 1×10^6 cells/mL in binding buffer with 50 μL/mL FITC Annexin V and 50 μL/mL PI. The cells were subsequently incubated for 15 min at room temperature and then were analyzed by Gallios flow cytometer (Beckman Coulter, USA). The cell's inner mitochondrial membrane potential (Δψm) was detected by flow cytometry using MitoScreen JC-1 staining kit (BD Biosciences) and was performed as previously described.[46] Flow cytometry data were analyzed using FlowJo 7.6 software (TreeStar, USA).

Caspase-9 or Caspase-3 Activity Assays

Activity of caspase-9 or caspase-3 was analyzed by spectrophotometry using a caspase-9 colorimetric assay kit or caspase-3 colorimetric assay kit (Keygen, China) and was performed as previously described.[25] The absorbance was measured at 405 nm, and

bicinchoninic acid (BCA) protein quantitative analysis was used as the reference to normal each experiment groups.

Tumor Xenografts

The 6-week-old BALB/c-nu mice were randomly divided into two groups (n = 5 per group). 1×10^6 SK-OV-3 cells per mouse were inoculated subcutaneously into the inguinal folds of the nude mice. After 12 days of cell inoculation, the mice were injected intraperitoneally with 2 mg/kg cisplatin every 5 days for 3 weeks. Tumor volume was determined using an external caliper and calculated using the equation $(L \times W^2)/2$. On day 42, animals were euthanized, and the tumors were excised, weighed, and stored in liquid nitrogen tanks.

Luciferase Assay

Cells (4×10^4) were seeded in triplicate in 24-well plates and cultured for 24 h, and the luciferase reporter assay was performed as previously described.[47] Cells were transfected with 100 ng TOP-flash or FOP-flash luciferase reporter plasmid, plus 5 ng pRL-TK Renilla plasmid (E2241; Promega) using Lipofectamine 3000 (Invitrogen) according to the manufacturer's recommendation. Luciferase and Renilla signals were measured 36 h after transfection using a dual luciferase reporter assay kit (Promega) according to the manufacturer's protocol.

Agent, Plasmid, and Transfection

S33Y and pcDNA3-S33Y β-catenin were purchased from Addgene (#19286). The reporter plasmids containing wild-type (CCTTT GATC; TOPflash, #12456, Addgene) or mutated (CCTTTGGCC; FOPflash, #12457, Addgene) T cell factor/lymphoid enhance factor (TCF/LEF) DNA binding sites were purchased from Upstate Biotechnology. Knockdown of endogenous LGR6 was performed by cloning two short hairpin RNA (shRNA) oligonucleotides into the pSUPER-puro-retro vector (OligoEngine, Seattle, WA, USA). Two separate shRNA fragments of LGR6 are listed in Table S5. Plasmid transfection was performed according to the protocol of Lipofectamine 3000 (Life Technologies).

Patients and Tumor Tissues

Six fresh ovarian cancer tissues and four normal ovarian epithelial tissues, as well as individual 294 paraffin-embedded, archived ovarian cancer tissues and 40 normal ovarian epithelial tissues were obtained during surgery at from the Clinical Biobank of Collaborative Innovation Center for Medical Molecular Diagnostics of Guangdong Province, the Affiliated Jiangmen Hospital of Sun Yat-sen University (Guangdong, China) between January 2008 and December 2017 (Tables S1, S2, and S6). Patients were diagnosed based on clinical and pathological evidence, and the specimens were immediately snap-frozen and stored in liquid nitrogen tanks. For the use of these clinical materials for research purposes, prior patients' consents and approval from the Institutional Research Ethics Committee were obtained.

Statistical Analysis

All values are presented as means ± SD. Significant differences were determined using GraphPad 5.0 software (USA). Student's t test was used to determine statistical differences between two groups. One-way ANOVA was used to determine statistical differences between multiple testing. The chi-square test was used to analyze the relationship between LGR6 expression and clinicopathological characteristics. Survival curves were plotted using the Kaplan-Meier method and compared by log-rank test. $p < 0.05$ was considered significant. All the experiments were repeated three times.

SUPPLEMENTAL INFORMATION

Supplemental Information can be found online at https://doi.org/10.1016/j.omto.2019.04.002.

AUTHOR CONTRIBUTIONS

Y.W. developed ideas and drafted the manuscript. X.R., A.L., and M.Z. conducted the experiments and contributed to the analysis of data. J.W., W.Z., and Y.R. contributed to the analysis of data. W.L., M.L., and X.Q. conducted the experiments. G.C., R.L., Y.L., and Q.L. contributed to the analysis of data and revised the manuscript. X.Z. and D.R. edited the manuscript. All authors contributed to revision of the manuscript and approved the final version for publication.

CONFLICTS OF INTEREST

The authors declare no competing interests.

ACKNOWLEDGMENTS

This study was supported by the grants from the National Natural Science Foundation of China (81702917 and 81802918), the Science and Technology Project of Guangdong Province (2018A030310007), the Medical Science Foundation of Guangdong Province (A2017103), the Science Foundation of Guangdong Province Bureau of Traditional Chinese Medicine (20181273), and the Medical Science Foundation of Jiangmen Central Hospital (J201801 and D201801).

REFERENCES

1. Jemal, A., Siegel, R., Ward, E., Hao, Y., Xu, J., and Thun, M.J. (2009). Cancer statistics, 2009. CA Cancer J. Clin. *59*, 225–249.
2. Yamamoto, M., Suzuki, S., Togashi, K., Sanomachi, T., Seino, S., Kitanaka, C., and Okada, M. (2018). AS602801, an Anticancer Stem Cell Candidate Drug, Reduces Survivin Expression and Sensitizes A2780 Ovarian Cancer Stem Cells to Carboplatin and Paclitaxel. Anticancer Res. *38*, 6699–6706.
3. Mihanfar, A., Aghazadeh Attari, J., Mohebbi, I., Majidinia, M., Kaviani, M., Yousefi, M., and Yousefi, B. (2018). Ovarian cancer stem cell: A potential therapeutic target for overcoming multidrug resistance. J. Cell. Physiol. *234*, 3238–3253.
4. Ge, G., Zhou, C., Ren, Y., Tang, X., Wang, K., Zhang, W., Niu, L., Zhou, Y., Yan, Y., and He, J. (2016). Enhanced SLC34A2 in breast cancer stem cell-like cells induces chemotherapeutic resistance to doxorubicin via SLC34A2-Bmi1-ABCC5 signaling. Tumour Biol. *37*, 5049–5062.
5. Zhang, L., Sun, H., Zhao, F., Lu, P., Ge, C., Li, H., Hou, H., Yan, M., Chen, T., Jiang, G., et al. (2012). BMP4 administration induces differentiation of CD133+ hepatic cancer stem cells, blocking their contributions to hepatocellular carcinoma. Cancer Res. *72*, 4276–4285.
6. Janzen, D.M., Tiourin, E., Salehi, J.A., Paik, D.Y., Lu, J., Pellegrini, M., and Memarzadeh, S. (2015). An apoptosis-enhancing drug overcomes platinum resistance in a tumour-initiating subpopulation of ovarian cancer. Nat. Commun. *6*, 7956.
7. Hirst, J., Pathak, H.B., Hyter, S., Pessetto, Z.Y., Ly, T., Graw, S., Koestler, D.C., Krieg, A.J., Roby, K.F., and Godwin, A.K. (2018). Licofelone Enhances the Efficacy of

Paclitaxel in Ovarian Cancer by Reversing Drug Resistance and Tumor Stem-like Properties. Cancer Res. *78*, 4370–4385.

8. Westendorf, J.J., Kahler, R.A., and Schroeder, T.M. (2004). Wnt signaling in osteoblasts and bone diseases. Gene *341*, 19–39.

9. Goessling, W., North, T.E., Loewer, S., Lord, A.M., Lee, S., Stoick-Cooper, C.L., Weidinger, G., Puder, M., Daley, G.Q., Moon, R.T., and Zon, L.I. (2009). Genetic interaction of PGE2 and Wnt signaling regulates developmental specification of stem cells and regeneration. Cell *136*, 1136–1147.

10. Ren, D., Dai, Y., Yang, Q., Zhang, X., Guo, W., Ye, L., Huang, S., Chen, X., Lai, Y., Du, H., et al. (2019). Wnt5a induces and maintains prostate cancer cells dormancy in bone. J. Exp. Med. *216*, 428–449.

11. Cai, J., Guan, H., Fang, L., Yang, Y., Zhu, X., Yuan, J., Wu, J., and Li, M. (2013). MicroRNA-374a activates Wnt/β-catenin signaling to promote breast cancer metastasis. J. Clin. Invest. *123*, 566–579.

12. Yang, L., Tang, H., Kong, Y., Xie, X., Chen, J., Song, C., Liu, X., Ye, F., Li, N., Wang, N., and Xie, X. (2015). LGR5 Promotes Breast Cancer Progression and Maintains Stem-Like Cells Through Activation of Wnt/β-Catenin Signaling. Stem Cells *33*, 2913–2924.

13. Zhang, Y., Zhang, X., Huang, J., and Dong, Q. (2015). Wnt signaling regulation of stem-like properties in human lung adenocarcinoma cell lines. Med. Oncol. *32*, 157.

14. Jang, G.B., Kim, J.Y., Cho, S.D., Park, K.S., Jung, J.Y., Lee, H.Y., Hong, I.S., and Nam, J.S. (2015). Blockade of Wnt/β-catenin signaling suppresses breast cancer metastasis by inhibiting CSC-like phenotype. Sci. Rep. *5*, 12465.

15. Chen, M.W., Yang, S.T., Chien, M.H., Hua, K.T., Wu, C.J., Hsiao, S.M., Lin, H., Hsiao, M., Su, J.L., and Wei, L.H. (2017). The STAT3-miRNA-92-Wnt Signaling Pathway Regulates Spheroid Formation and Malignant Progression in Ovarian Cancer. Cancer Res. *77*, 1955–1967.

16. Mariya, T., Hirohashi, Y., Torigoe, T., Tabuchi, Y., Asano, T., Saijo, H., Kuroda, T., Yasuda, K., Mizuuchi, M., Saito, T., and Sato, N. (2016). Matrix metalloproteinase-10 regulates stemness of ovarian cancer stem-like cells by activation of canonical Wnt signaling and can be a target of chemotherapy-resistant ovarian cancer. Oncotarget *7*, 26806–26822.

17. Hsu, S.Y., Kudo, M., Chen, T., Nakabayashi, K., Bhalla, A., van der Spek, P.J., van Duin, M., and Hsueh, A.J. (2000). The three subfamilies of leucine-rich repeat-containing G protein-coupled receptors (LGR): identification of LGR6 and LGR7 and the signaling mechanism for LGR7. Mol. Endocrinol. *14*, 1257–1271.

18. Leushacke, M., and Barker, N. (2012). Lgr5 and Lgr6 as markers to study adult stem cell roles in self-renewal and cancer. Oncogene *31*, 3009–3022.

19. Xie, Y., Sundstrom, A., Maturi, N.P., Tan, E.J., Marinescu, V.D., Jarvius, M., Tirfing, M., Jin, C., Chen, L., Essand, M., et al. (2019). LGR5 promotes tumorigenicity and invasion of glioblastoma stem-like cells and is a potential therapeutic target for a subset of glioblastoma patients. J. Pathol. *247*, 228–240.

20. Xu, P., Dang, Y., Wang, L., Liu, X., Ren, X., Gu, J., Liu, M., Dai, X., and Ye, X. (2016). Lgr4 is crucial for skin carcinogenesis by regulating MEK/ERK and Wnt/β-catenin signaling pathways. Cancer Lett. *383*, 161–170.

21. Tan, B., Shi, X., Zhang, J., Qin, J., Zhang, N., Ren, H., Qian, M., Siwko, S., Carmon, K., Liu, Q., et al. (2018). Inhibition of Rspo-Lgr4 Facilitates Checkpoint Blockade Therapy by Switching Macrophage Polarization. Cancer Res. *78*, 4929–4942.

22. de Lau, W., Barker, N., Low, T.Y., Koo, B.K., Li, V.S., Teunissen, H., Kujala, P., Haegebarth, A., Peters, P.J., van de Wetering, M., et al. (2011). Lgr5 homologues associate with Wnt receptors and mediate R-spondin signalling. Nature *476*, 293–297.

23. Carmon, K.S., Gong, X., Lin, Q., Thomas, A., and Liu, Q. (2011). R-spondins function as ligands of the orphan receptors LGR4 and LGR5 to regulate Wnt/beta-catenin signaling. Proc. Natl. Acad. Sci. USA *108*, 11452–11457.

24. Gong, X., Carmon, K.S., Lin, Q., Thomas, A., Yi, J., and Liu, Q. (2012). LGR6 is a high affinity receptor of R-spondins and potentially functions as a tumor suppressor. PLoS ONE *7*, e37137.

25. Ren, D., Lin, B., Zhang, X., Peng, Y., Ye, Z., Ma, Y., Liang, Y., Cao, L., Li, X., Li, R., et al. (2017). Maintenance of cancer stemness by miR-196b-5p contributes to chemoresistance of colorectal cancer cells via activating STAT3 signaling pathway. Oncotarget *8*, 49807–49823.

26. Al-Ejeh, F., Smart, C.E., Morrison, B.J., Chenevix-Trench, G., López, J.A., Lakhani, S.R., Brown, M.P., and Khanna, K.K. (2011). Breast cancer stem cells: treatment resistance and therapeutic opportunities. Carcinogenesis *32*, 650–658.

27. Tao, J., Zhang, R., Singh, S., Poddar, M., Xu, E., Oertel, M., Chen, X., Ganesh, S., Abrams, M., and Monga, S.P. (2017). Targeting β-catenin in hepatocellular cancers induced by coexpression of mutant β-catenin and K-Ras in mice. Hepatology *65*, 1581–1599.

28. Guinot, A., Oeztuerk-Winder, F., and Ventura, J.J. (2016). miR-17-92/p38α Dysregulation Enhances Wnt Signaling and Selects Lgr6+ Cancer Stem-like Cells during Lung Adenocarcinoma Progression. Cancer Res. *76*, 4012–4022.

29. van de Glind, G.C., Rebel, H.G., Out-Luiting, J.J., Zoutman, W., Tensen, C.P., and de Gruijl, F.R. (2016). Lgr6+ stem cells and their progeny in mouse epidermis under regimens of exogenous skin carcinogenesis, and their absence in ensuing skin tumors. Oncotarget *7*, 86740–86754.

30. Garcia-Closas, M., Couch, F.J., Lindstrom, S., Michailidou, K., Schmidt, M.K., Brook, M.N., Orr, N., Rhie, S.K., Riboli, E., Feigelson, H.S., et al. (2013). Genome-wide association studies identify four ER negative-specific breast cancer risk loci. Nat. Genet. *45*, 392–398, 398e1–e2.

31. Schindler, A.J., Watanabe, A., and Howell, S.B. (2017). LGR5 and LGR6 in stem cell biology and ovarian cancer. Oncotarget *9*, 1346–1355.

32. Walker, F., Zhang, H.H., Odorizzi, A., and Burgess, A.W. (2011). LGR5 is a negative regulator of tumourigenicity, antagonizes Wnt signalling and regulates cell adhesion in colorectal cancer cell lines. PLoS ONE *6*, e22733.

33. Wu, C., Qiu, S., Lu, L., Zou, J., Li, W.F., Wang, O., Zhao, H., Wang, H., Tang, J., Chen, L., et al. (2014). RSPO2-LGR5 signaling has tumour-suppressive activity in colorectal cancer. Nat. Commun. *5*, 3149.

34. Blaas, L., Pucci, F., Messal, H.A., Andersson, A.B., Josue Ruiz, E., Gerling, M., Douagi, I., Spencer-Dene, B., Musch, A., Mitter, R., et al. (2016). Lgr6 labels a rare population of mammary gland progenitor cells that are able to originate luminal mammary tumours. Nat. Cell Biol. *18*, 1346–1356.

35. Snippert, H.J., Haegebarth, A., Kasper, M., Jaks, V., van Es, J.H., Barker, N., van de Wetering, M., van den Born, M., Begthel, H., Vries, R.G., et al. (2010). Lgr6 marks stem cells in the hair follicle that generate all cell lineages of the skin. Science *327*, 1385–1389.

36. Oeztuerk-Winder, F., Guinot, A., Ochalek, A., and Ventura, J.J. (2012). Regulation of human lung alveolar multipotent cells by a novel p38α MAPK/miR-17-92 axis. EMBO J. *31*, 3431–3441.

37. Ren, W., Lewandowski, B.C., Watson, J., Aihara, E., Iwatsuki, K., Bachmanov, A.A., Margolskee, R.F., and Jiang, P. (2014). Single Lgr5- or Lgr6-expressing taste stem/progenitor cells generate taste bud cells ex vivo. Proc. Natl. Acad. Sci. USA *111*, 16401–16406.

38. Huang, P.Y., Kandyba, E., Jabouille, A., Sjolund, J., Kumar, A., Halliwill, K., McCreery, M., DelRosario, R., Kang, H.C., Wong, C.E., et al. (2017). Lgr6 is a stem cell marker in mouse skin squamous cell carcinoma. Nat. Genet. *49*, 1624–1632.

39. Ren, D., Yang, Q., Dai, Y., Guo, W., Du, H., Song, L., and Peng, X. (2017). Oncogenic miR-210-3p promotes prostate cancer cell EMT and bone metastasis via NF-κB signaling pathway. Mol. Cancer *16*, 117.

40. Dai, Y., Ren, D., Yang, Q., Cui, Y., Guo, W., Lai, Y., Du, H., Lin, C., Li, J., Song, L., and Peng, X. (2017). The TGF-β signalling negative regulator PICK1 represses prostate cancer metastasis to bone. Br. J. Cancer *117*, 685–694.

41. Ren, D., Wang, M., Guo, W., Zhao, X., Tu, X., Huang, S., Zou, X., and Peng, X. (2013). Wild-type p53 suppresses the epithelial-mesenchymal transition and stemness in PC-3 prostate cancer cells by modulating miR-145. Int. J. Oncol. *42*, 1473–1481.

42. Guo, W., Ren, D., Chen, X., Tu, X., Huang, S., Wang, M., Song, L., Zou, X., and Peng, X. (2013). HEF1 promotes epithelial mesenchymal transition and bone invasion in prostate cancer under the regulation of microRNA-145. J. Cell. Biochem. *114*, 1606–1615.

43. Li, X., Liu, F., Lin, B., Luo, H., Liu, M., Wu, J., Li, C., Li, R., Zhang, X., Zhou, K., and Ren, D. (2017). miR-150 inhibits proliferation and tumorigenicity via retarding G1/S

phase transition in nasopharyngeal carcinoma. Int. J. Oncol. Published online March 10, 2017. https://doi.org/10.3892/ijo.2017.3909.

44. Goodell, M.A. (2005). Stem cell identification and sorting using the Hoechst 33342 side population (SP). Curr. Protoc. Cytom. *33*, 9.18.1–9.18.11.

45. Ren, D., Wang, M., Guo, W., Huang, S., Wang, Z., Zhao, X., Du, H., Song, L., and Peng, X. (2014). Double-negative feedback loop between ZEB2 and miR-145 regulates epithelial-mesenchymal transition and stem cell properties in prostate cancer cells. Cell Tissue Res. *358*, 763–778.

46. Zhang, X., Ren, D., Wu, X., Lin, X., Ye, L., Lin, C., Wu, S., Zhu, J., Peng, X., and Song, L. (2018). miR-1266 Contributes to Pancreatic Cancer Progression and Chemoresistance by the STAT3 and NF-κB Signaling Pathways. Mol. Ther. Nucleic Acids *11*, 142–158.

47. Zhang, X., Zhang, L., Lin, B., Chai, X., Li, R., Liao, Y., Deng, X., Liu, Q., Yang, W., Cai, Y., et al. (2017). Phospholipid Phosphatase 4 promotes proliferation and tumorigenesis, and activates Ca^{2+}-permeable Cationic Channel in lung carcinoma cells. Mol. Cancer *16*, 147.

OMTO, Volume *14*

Supplemental Information

Silencing LGR6 Attenuates Stemness and Chemoresistance via Inhibiting Wnt/β-Catenin Signaling in Ovarian Cancer

Xiaohong Ruan, Aibin Liu, Meigong Zhong, Jihong Wei, Weijian Zhang, Yingrou Rong, Wanmin Liu, Mingwei Li, Xingrong Qing, Gaowen Chen, Ronggang Li, Yuehua Liao, Qiongru Liu, Xin Zhang, Dong Ren, and Yifeng Wang

Supplemental Table 1. The basic information of 40 patients with normal ovarian control for LGR6 immunohistochemical staining analysis.

		Cases (n, %)
Age	<50	11 (27.5)
	≥50	29 (72.5)
Type of diseases	Malignant lesions on the contralateral ovary	1 (2.5)
	Non-malignant lesions on the contralateral ovary	13 (32.5)
	Malignant lesions without ovary	5 (12.5)
	Non-malignant lesions without ovary	21 (52.5)

Note: (1) Malignant lesions on the contralateral ovary included endometrioid adenocarcinoma. (2) Non-malignant lesions on the contralateral ovary included simple cyst, Brenner tumor, serous cystadenoma and fibrothecoma. (3) Malignant lesions without ovary included breast cancer, cervical cancer and uterus cancer. (4) Non-malignant lesions without ovary included cervical intraepithelial neoplasia, mesosalpinx cyst, uterine fibroids, endometrial hyperplasia with atypia and prolapse of uterus.

Supplemental Table 2. The basic information of 294 patients with epithelial ovarian cancer for LGR6 immunohistochemical staining analysis.

		Cases (n)	Percentage (%)
Age	<50	128	43.5
	≥50	166	56.5
Histologic	Low grade serous adenocarcinoma	27	9.2
	High grade serous adenocarcinoma	162	55.1
	Mucinous adenocarcinoma	42	14.3
	Endometrioid adenocarcinoma	29	9.9
	Clear cell adenocarcinoma	16	5.4
	Other	18	6.1
FIGO stage	Stage I	90	30.6
	Stage II	49	16.7
	Stage III	130	44.2
	Stage IV	12	4.1
	NA	13	4.4
Progression status (in 3 y)	Positive	91	31.0
	Negative	54	18.4
	NA	149	50.7
First chemotherapy response (at 6 m)	Sensitivity	63	21.4
	Resistance	30	10.2
	NA	201	68.4

Note: (1) Other histologic types included mixed adenocarcinoma, serous-mucinous adenocarcinoma, undifferentiated carcinoma, carcinosarcoma and non-specific type adenocarcinoma. (2) NA: Not available; FIGO: The International Federation of Gynecology and Obstetrics.

Supplemental Table 3. The relationship between LGR6 IHC expression level and clinical pathological characteristics in 294 patients with epithelial ovarian cancer.

Parameters	Number of cases	LGR6 IHC expression		P values
		Low	High	
Age				
<50	128	55	63	0.705
≥50	166	86	90	
Histologic				
Type I	114	68	46	<0.001
Type II	164	62	102	
FIGO stage				
I – II	139	84	55	<0.001
III - IV	142	49	93	
Progression status (in 3 y)				
Positive	91	20	70	0.004
Negative	54	24	29	
First chemotherapy response (at 6 m)				
Sensitivity	63	39	24	<0.001
Resistance	30	2	28	

Note: (1) Histologic type I included low grade serous adenocarcinoma, mucinous adenocarcinoma, endometrioid adenocarcinoma and clear cell adenocarcinoma. Histologic type II included high grade serous adenocarcinoma and undifferentiated carcinoma. (2) IHC: Immunohistochemistry; FIGO: The International Federation of Gynecology and Obstetrics.

Supplemental Table 4. A list of primers used in the reactions for real-time PCR.

Gene name		Sequence
ABCG2	forward	5'- ATGAACACACATGTGCAACCATC -3'
	reverse	5'- CACAGAAACACAACACTTGGCTGTA -3'
CCND1	forward	5'- CGTGGCCTCTAAGATGAAGG -3'
	reverse	5'- CCACTTGAGCTTGTTCACCA -3'
CDH1	forward	5'- ATGAGTGTCCCCCGGTATCT -3'
	reverse	5'- GGCTGTGGGGTCAGTATCAG -3'
DKK3	forward	5'- GTGCTGTGCCTTCCAGAGA -3'
	reverse	5'- GCTCTAGCTCCCAGGTGATG -3'
GAPDH	forward	5'- GCACCGTCAAGGCTGAGAAC -3'
	reverse	5'- TGGTGAAGACGCCAGTGGA -3'
KLF4	forward	5'- GTTCCCATCTCAAGGCACAC -3'
	reverse	5'- ACGGTAGTGCCTGGTCAGTT -3'
LEF1	forward	5'- CCATCCCGAGAACATCAAAT -3'
	reverse	5'- CCTGGAGAAAAGTGCTCGTC -3'
LGR4	forward	5'- TGATGCAGCAAATGTCACAA -3'
	reverse	5'- GGGCTTAAAAGCACCTGTTG -3'
LGR5	forward	5'- TGAATTTGGCTTGGAACAAA -3'
	reverse	5'- AAAGACGACAGGAGGTTGGA -3'
LGR6	forward	5'- AAGGGAACCTTGCTCTCTCC -3'
	reverse	5'- CATCCCATAGGGACAGCACT -3'
MMP7	forward	5'- TGTATGGGGAACTGCTGACA -3'
	reverse	5'- ATCTCCTCCGAGACCTGTCC -3'
NANOG	forward	5'- TCCAACATCCTGAACCTCAGCTA -3'
	reverse	5'- AGTCGGGTTCACCAGGCATC -3'
OCT4	forward	5'- TGAAGCTGGAGAAGGAGAAGCTG -3'
	reverse	5'- GCAGATGGTCGTTTGGCTGA -3'
SOX2	forward	5'- GTGAGCGCCCTGCAGTACAA -3'
	reverse	5'- GCGAGTAGGACATGCTGTAGGTG -3'
TCF1	forward	5'- ACCTGTCCCAACACCTCAAC -3'
	reverse	5'- CTCATCACCTGTGGGCTCTT -3'
VEGFA	forward	5'- AGACCCTGGTGGACATCTTC -3'
	reverse	5'- ATCCGCATAATCTGCATGGT -3'

Supplemental Table 5. A list of primers used in the reactions for clone PCR.

Gene name		Sequence
shLGR6:#1	forward	5'- CCGG-GAGGGAAGTAAAGACAGTGAA-CTCGAG-TTCACTGTCTTTACTTCCCTC-TTTTTG -3'
	reverse	5'-CAAAAA-GAGGGAAGTAAAGACAGTGAA-CTCGAG-TTCACTGTCTTTACTTCCCTC-CCGG-3'
shLGR6#2	forward	5'- CCGG-GCATTCCAGTACCTGCCTAAA-CTCGAG-TTTAGGCAGGTACTGGAATGC-TTTTTG -3'
	reverse	5'- CAAAAA-GCATTCCAGTACCTGCCTAAA-CTCGAG-GCATTCCAGTACCTGCCTAAA-CCGG-3'

Supplemental Table 6. The basic information of 6 epithelial ovarian cancer patients and 4 normal ovarian control patients for LGR6 mRNA and protein expression analysis.

		Cases (n, %)	
		EOC	NC
Age	≤50	3 (50.0)	2 (50.0)
	>50	3 (50.0)	2 (50.0)
Disease	Low grade serous adenocarcinoma	1 (16.7)	0
	High grade serous adenocarcinoma	2 (33.3)	0
	Mucinous adenocarcinoma	1 (16.7)	0
	Endometrioid adenocarcinoma	1 (16.7)	0
	Clear cell adenocarcinoma	1 (16.7)	0
	Cervical adenocarcinoma	0	2 (50.0)
	Endometrial adenocarcinoma	0	1 (50.0)
	Cervical squamous carcinoma	0	1 (50.0)

Note: EOC: epithelial ovarian cancer; NC: normal control.
